# How Did the COVID-19 Pandemic Affect Migrant Populations in Lisbon, Portugal? A Study on Perceived Effects on Health and Economic Condition

**DOI:** 10.3390/ijerph19031786

**Published:** 2022-02-04

**Authors:** Ana Gama, João Victor Rocha, Maria J. Marques, Sofia Azeredo-Lopes, Ana Rita Pedro, Sónia Dias

**Affiliations:** 1 Public Health Research Centre, NOVA National School of Public Health, Universidade NOVA de Lisboa, 1600-560 Lisboa, Portugal; anafgama@gmail.com (A.G.); jv.rocha@ensp.unl.pt (J.V.R.); mj.marques@ensp.unl.pt (M.J.M.); rita.pedro@ensp.unl.pt (A.R.P.); 2 Comprehensive Health Research Centre (CHRC), Universidade NOVA de Lisboa, 1169-056 Lisboa, Portugal; sofia.azeredo@nms.unl.pt; 3 EpiDoC Unit, CEDOC, NOVA Medical School, Universidade NOVA de Lisboa, 1169-056 Lisboa, Portugal; 4 Statistics and Operational Research Department, Sciences Faculty, University of Lisbon, 1749-016 Lisboa, Portugal

**Keywords:** migrants, COVID-19 pandemic, health disparities, socioeconomic inequalities, public health

## Abstract

Increasing evidence on the effects of the COVID-19 pandemic suggests that its social and health impacts are being disproportionately shouldered by socioeconomically disadvantaged populations, including migrants. Knowledge of how these populations are experiencing the COVID-19 crisis is scarce. We examined the effects of the pandemic on the perceived individual financial situation and health condition of migrants in Lisbon, Portugal, and described the most affected subgroups. A cross-sectional survey was conducted with a diverse community-based sample of 1126 migrants. A worsening of their financial situation since the pandemic was reported by 55.6% of the participants and a worsening of their health condition by 19.9%. A worsened financial situation was most often reported by those ≥45 years old and with a lower income (<EUR 650). Likewise, a worsened health condition was most often reported by older and lower-income migrants, as well as by women and those with a lower level of education. Migration-related factors such as length of stay and migration status were not associated with worsened health conditions. Socioeconomic characteristics appear to be more important when assessing differences in perceived effects of the pandemic among migrants. The social impact of the pandemic both exacerbates economic and gender inequalities and may lead to worse health conditions within the population in the medium and long terms.

## 1. Introduction

The COVID-19 pandemic brought new challenges to societies and their health systems. In order to contain the spread of the virus and face this public health emergency, several preventive measures were implemented, resulting in the suspension of various activities in society and general lockdowns that strongly affected the lives of citizens. The documented experience from previous economic crises and the first evidence available on the effects of the COVID-19 pandemic suggest that its social and health impacts are being disproportionately shouldered by socioeconomically disadvantaged populations. This includes particular groups of migrants, who are likely to experience financial and material deprivation, face difficulties in accessing health services and are at increased risk of social exclusion [1,2,3].

Assessing to what extent COVID-19 has affected migrant populations has been challenging. Evidence on the initial effects of the pandemic is still limited and mainly based on official statistics from diverse sources, where data on country of birth, nationality or migration status are often not registered [4]. In most countries for which data are available, analysis reveals a disproportionate impact of the pandemic on migrants, especially in Southern European countries, but also in Ireland, Norway and Sweden [4]. For instance, recent statistics from health authorities in Barcelona show that the coronavirus outbreak has apparently affected migrant communities and lower-income neighborhoods the hardest [5]. In terms of the social impact of the pandemic, OECD calculations based on data from national employment statistics indicate that most OECD countries experienced increases in the unemployment rate for both native-born and immigrant residents, with a much larger increase for the latter [4]. In a nationwide survey in the UK, results showed that Black, Asian and minority ethnic migrants had a lower level of employment protection, were more likely to experience income loss and faced increased financial hardship during the pandemic, compared with UK-born white British residents [6]. Certain subgroups are particularly underserved; often, those at the margins of society, such as recently arrived undocumented migrants with reduced social support and resources to cope with hardship, may face more severe adversity during the pandemic. In these circumstances, the health needs of these populations are even more difficult to address. In addition, the vast literature on social determinants of health supports that the deterioration of the socioeconomic condition during the COVID-19 pandemic will most likely exacerbate health vulnerability among migrants, with negative effects on health outcomes [7,8].

The impact of the pandemic on migrant populations has been heavily stressed by editorial texts in the scientific literature and technical reports from international agencies [9,10,11,12,13]. Nevertheless, there is scarce empirical evidence from research studies, including in Portugal, on how these populations are experiencing the COVID-19 crisis and the perceived effects on their socioeconomic and health conditions [6,9]. This knowledge, particularly regarding the most socially vulnerable and understudied subgroups, is essential for designing effective evidence-based migrant-sensitive policies and strategies that ensure that no communities are left behind. This study aimed to examine the effects of the COVID-19 pandemic on the perceived financial situation and health conditions among migrant communities in Lisbon, Portugal, and describe the most affected subgroups.

## 2. Materials and Methods

This cross-sectional study consisted of a survey conducted with a community-based sample of migrants in the Lisbon district, the urban area where most of the foreign-born population in the country resides [14]. In 2019, there were 576,540 foreign citizens with an authorized residence permit in Portugal, and 50% of them resided in the Lisbon Metropolitan Area, comprising 10.4% of the total population in this region [15]. The most common nationalities are Brazilian, Cape Verdean, Angolan and Guinean, with Chinese, Indian and Nepali nationalities on the rise [14].

According to the definition of the International Organization for Migration (IOM), a “migrant” is “a person who moves away from his or her place of usual residence, whether within a country or across an international border, temporarily or permanently, and for a variety of reasons” [16]. The inclusion criteria included: being ≥18 years old, speaking Portuguese, English, Arabic, Bengali, Hindi, Mandarin or Nepali, residing in Portugal for ≤10 years and currently residing in the Lisbon Metropolitan Area, regardless of migration status (i.e., regular or irregular). Migrants were also included based on their country of origin, with the criterion of being born in a Portuguese-speaking African country, Brazil or a Middle Eastern or Asian country (i.e., the countries with greater representation in the migrant population of Portugal). Migrants who originated from European countries were not included due to their distinct socioeconomic status (most have higher education, highly qualified jobs and a high income) and the advantageous conditions of their arrival and permanence in the country [17].

There was no sampling frame which allowed us to draw a representative sample of migrants in the Lisbon Metropolitan Area while also including the most socially vulnerable groups, who are usually the most understudied and underserved, such as undocumented migrants. Stakeholders, such as non-governmental and governmental organizations and migrant associations that work in proximity with migrant communities, were engaged in the study from the start. These partners collaborated in publicizing the study within the communities and their networks and served as recruitment sites, where attendees were approached and invited to participate in the study. Several informal leaders of the migrant communities were invited to collaborate as recruiters of potential participants within their social networks, and as interpreters. This approach allowed us to reach and obtain information from a total of 1126 migrants.

Data were collected using a questionnaire applied through an interview by trained bilingual researchers, to ensure inclusion of people with low educational levels or who experience difficulties with self-administration. The questionnaire included items on demographic, socioeconomic and migration-related characteristics (i.e., sex, age, education level, monthly income before the COVID-19 outbreak, length of stay in Portugal, migration status). Participants were also asked about the perceived impact of the COVID-19 pandemic using the questions “Since the coronavirus crisis, how is your financial situation?” and “Since the coronavirus crisis, how is your health condition?”, with response options being “Got worse”, “The same as before” and “Got better”. The instrument was pre-tested and was made available in Portuguese, English, Arabic, Bengali, Hindi, Mandarin and Nepali. The application of the questionnaire lasted approximately 10 min. The study was approved by the Ethical Committee of NOVA Medical School (nr.142/2019/CEFCM). Informed consent was obtained from all participants.

### Data Analysis

Descriptive analysis was performed regarding absolute and relative frequencies of the variables for the whole sample and subgroups, based on perceived change in the individual financial situation and perceived change in the individual health condition since the COVID-19 pandemic. For analysis, the response options of the independent variables were dichotomized into “Got worse” and “The same as before/got better”. Although it was our initial intention to fit an ordinal logistic regression model and therefore to be able to include the three distinct levels (“worse”, “same” and “better”), the low numbers of individuals within the “better” category made quite an unbalanced sample size, affecting the precision of the model parameter estimates and also making it difficult to check the proportional odds assumption. Since the focus of the study is on how several factors might contribute to a “worse” outcome, we decided that the best option was to combine the “same” and “better” categories and fit a logistic regression model. Logistic regression models were obtained to estimate the crude odds ratios (ORs) and adjusted odds ratios (aORs), with corresponding 95% confidence intervals (CI) of the factors associated with perceived worse financial situation and worse health condition since the COVID-19 pandemic. A significance level of 0.05 was used throughout. Data analysis was performed using the R software version 4.0.2 (R Foundation for Statistical Computing, Vienna, Austria, 22 June 2020).

## 3. Results

Participants’ sociodemographic and migration-related characteristics are shown in Table 1. Of the 1126 respondents, 53.4% were women, most were between 26 and 45 years old (66%), 42.5% had secondary education and 26.2% had a lower education level. Around 65% of participants had a monthly income below EUR 650 before the COVID-19 outbreak. More than 80% had resided in Portugal for over one year. Overall, 9.3% of the participants were currently undocumented, and 90.7% were documented or in the process of regularization. Around 49% were born in a Portuguese-speaking African country, 29.5% in a Middle Eastern or South Asian country and 21.6% in Brazil.

Overall, 55.6% of the participants reported that their financial situation had worsened since the COVID-19 pandemic. Table 2 shows the adjusted OR estimates obtained by the logistic regression model, with the perceived change in the individual financial situation as the dependent variable. Higher odds of a perceived worse financial situation were found among older participants (>45 years: aOR = 2.01, CI 95% 1.25–3.25; 26–45 years: aOR = 1.63, CI 95% 1.12–2.37) and those with a lower income (aOR = 2.81, CI 95% 2.13–3.72). In the univariate analysis, the risk of a perceived worse financial situation since the pandemic was significantly higher among undocumented migrants (OR = 1.71, CI 95% 1.08–2.74) (Supplementary Materials Table S1), although it was not found to be significant in the adjusted model.

For approximately 20% of the participants, their individual health condition had worsened since the COVID-19 pandemic. Table 3 shows the adjusted OR estimates of the logistic regression model, with the perceived change in health condition as the dependent variable. The risk of perceiving a deteriorating health condition since the COVID-19 pandemic was significantly higher among women (aOR = 1.58, CI 95% 1.13–2.20), those older than 45 years (aOR = 1.78, CI 95% 1.02–3.16), with lower levels of education (basic education: aOR = 1.57, CI 95% 1.01–2.47; secondary education: aOR = 1.51, CI 95% 1.01–2.29) and with a monthly income lower than EUR 650 (aOR = 1.69, CI 95% 1.18–2.44). Migration-related factors such as length of stay in Portugal and migration status were not associated with worse perceived health conditions (*p* value > 0.05).

## 4. Discussion

To our knowledge, this is one of the few studies to examine the financial and health effects of the COVID-19 pandemic, as reported by migrants themselves. We found that these communities were highly impacted by the pandemic, and particularly the most socially vulnerable subgroups. The perception that the individual financial situation had worsened since the COVID-19 pandemic was stronger among people aged 45 years or older and with a lower monthly income (<EUR 650). Likewise, older and lower-income participants, in addition to women and those with lower levels of education, were the ones who most frequently reported worsening of their health condition.

These findings corroborate other studies which indicate that social and economic vulnerability is associated with a worse impact of the pandemic, both among migrant populations [4,5,9,18] and the population in general [19,20]. Monthly income was the most important variable in the models for both outcomes, with migrants with a lower income being almost three times more likely to report a worse financial situation and almost two times more likely to report a worse health condition. Although the pandemic affects all citizens, its impact is not equal for all: it has a social gradient that further accentuates the existing social and health inequalities [21,22].

It is worth mentioning that migration-related characteristics were not associated with perceived changes in health and financial conditions, which was the case of length of stay. This may be explained by the fact that all migrants included in the study resided in Portugal for ≤10 years, so this variable may not be helpfully discriminative. In addition, since the migrants included in the study resided for less time in Portugal, they may have reduced access to social protection and support measures, which intensifies the socioeconomic disadvantage and translates into lower income. Overall, the study results indicate that socioeconomic characteristics appear to be more important when assessing differences in the perceived effects of the pandemic among migrant populations. These findings are in line with those found for the general Portuguese population in the community-based survey “COVID-19 Barometer: Social Opinion”. In this study, a stronger impact of the COVID-19 pandemic on mental health and anxiety was found among persons with a basic educational level and those with a low income [23]. This suggests that socioeconomic position is on the basis of the asymmetric effects of the pandemic, for migrant and non-migrant populations. Many migrant groups face situations of socioeconomic fragility (e.g., low income, unemployment, lack of social support and resources), making them particularly vulnerable amongst those most affected by the pandemic. Indeed, our findings show that undocumented migrants were the group that most reported a worsening of their financial situation in the univariate analysis, indicating that extra attention to this group is needed. This finding is consistent with evidence showing an association between undocumented status and poorer socioeconomic condition [24,25]. Nevertheless, the fact that this association was not significant in the multivariate analysis limits the robustness of this finding and must be considered. It can be explained by the low number of undocumented migrants in the sample, despite the efforts to reach this understudied group, and a potential for collinearity cannot be excluded. Future research that reaches higher numbers of undocumented migrants will be valuable to further understand the disparities in the pandemic impact across migrant groups.

Female migrants reported a higher risk of perceiving a worse health condition since the COVID-19 pandemic. In other research on the effects of the pandemic, women in general reported a worse impact on their health, well-being and daily life [23], and higher self-perceived risk for worse COVID-19 outcomes [26]. Research on differences in perceived health by gender has shown that women consider themselves to be more vulnerable to illness, give a lower assessment of their current and past health state, are more concerned about health issues and demand more health care services than men [27,28,29]. Authors have referred to the symptom perception model to explain this trend: increased somatization and symptom reporting among women is associated with their heightened negative affect and sensitivity to internal symptoms, and higher selective attention to the body, and this may help explain our findings [30]. In addition, studies in different countries have found evidence of gender inequalities during COVID-19, showing that women had their employment status disproportionally affected by the pandemic when compared to men (e.g., increased transitions to unemployment, working hour reductions, changes to working from home) [31,32,33]. The available evidence also suggests that during the pandemic crisis, with the temporary closure of schools, in particular elementary schools, and the increased care needs among the elderly, the responsibility for informal care provision fell more heavily on women [34]. Women (migrant and non-migrant) constitute the majority of caregivers in health and social care facilities, in private households and in their own homes, and this has increased during the COVID-19 pandemic [35,36]. Increased workload and financial/job insecurity can have adverse effects on health, including mental health, especially among women, including migrants [34]. Indeed, studies indicate that the pandemic had a more deleterious effect on women’s mental health outcomes (e.g., anxiety, depressive symptoms), compared to males [37]. Evidence also shows that the pandemic has negatively impacted mental health across all migrant groups [24,37], in particular female migrants [38].

The proportion of people who reported a worse financial situation was higher compared to those reporting a worse health condition (55.6% vs. 19.9%). The survey was conducted in the second semester of 2020; thus, there may have been enough time for participants to note a worsening of their financial situation, but not enough time for effects on their health condition to become perceptible. 

The lack of association between migration status and perceived change in health condition may be partially explained by the fact that, in Portugal, all citizens are guaranteed the right to receive health care in the National Health Service (health center or hospital), regardless of their migration status, nationality or economic means. In addition, in response to the COVID-19 pandemic, the Portuguese government determined that all migrants with pending residence permit applications would be granted temporary residence status and have access to the same rights as all other citizens, including health and social support [10,39]. This governmental action may have mitigated some of the negative health effects of the pandemic and its response measures. However, it is fair to assume that there were still difficulties in getting or maintaining a remunerated job during the pandemic, as well as accessing social support measures (e.g., no possibility of collecting unemployment benefits due to the lack of a previous legal work contract), which can potentially affect health.

Our study has limitations that must be acknowledged. The sampling method used generated a non-probabilistic sample, thus limiting the ability to generalize findings to the broader migrant population. Nonetheless, the recruitment strategy used, through the collaboration of non-governmental and governmental organizations and migrant associations that work in proximity with migrant communities, allowed us to gather a large and diverse sample of migrants residing in the Lisbon Metropolitan Area, including different origin and socioeconomic groups, and to reach some of the most socially vulnerable, who are usually most understudied, particularly those who are undocumented. This study was not conducted in regions outside of the Lisbon area; nevertheless, to our knowledge, the characteristics of the migrant population in this area do not significantly differ from those living in the rest of the country. This study assessed perceptions of the pandemic impact, so responses may be influenced by personal characteristics, such as optimism or exaggeration, and may not reflect the actual situation. The fact that this study was based on self-reported data and the use of face-to-face interviews, to include people with lower levels of education, may have led to misreporting of financial and health effects of the pandemic. Nevertheless, all interviewers were systematically trained in interview techniques and ethical principles to minimize potential biases. In the worst-case scenario, our data likely underestimate the perceived impact of the pandemic among the studied population. Finally, the perceived impact of the pandemic was measured using three degrees (“worse”, “same” and “better” since the crisis), but no information was collected that would allow further differentiation within each degree.

## 5. Conclusions

Migrant populations face a range of vulnerabilities during non-pandemic times. The social and health impacts of the COVID-19 pandemic have disproportionally fallen on the most vulnerable overall. The social impact of the COVID-19 pandemic both exacerbates the existing economic and gender inequalities and may lead to a worsening of health conditions within the population. Therefore, it is important to design policies that integrate the health and social sectors to mitigate those inequalities. Despite the exceptional measures implemented during the pandemic crisis to assure social protection of citizens, more measures especially aimed at migrants should be taken to promote access to labor and social protection measures that mitigate the economic and social effects of the pandemic among those most vulnerable. Additionally, assuring that the information on the existing social support measures is broadly available, in diverse languages and in formats that are understandable and accessible to migrants, is valuable.

## Figures and Tables

**Table 1:** Characteristics of the participants.

	*N*	%
Sex (*n* = 1126)		
Women	601	53.4
Men	525	46.6
Age (*n* = 1125) ^a^		
18–25 years	182	16.2
26–45 years	743	66.0
>45 years	200	17.8
Education level (*n* = 1119)		
Basic education	293	26.2
Secondary education	476	42.5
Higher education	350	31.3
Monthly income before COVID-19 outbreak (*n* = 1100) ^b^		
<EUR 650	714	64.9
≥EUR 650	386	35.1
Length of stay in Portugal (*n* = 1125)		
<1 year	223	19.8
1 to 5 years	738	65.6
≥6 years	164	14.6
Migration status (*n* = 1111)		
Documented/in regularization	1008	90.7
Undocumented	103	9.3

^a^ The age groups were established considering different life stages that are often related to migration patterns. ^b^ The monthly income groups were established based on the average Portuguese gross minimum monthly wage for the data collection years (EUR 650).

**Table 2:** Characteristics of the participants by perceived change in their individual financial situation since the COVID-19 pandemic and factors associated with worse financial situation.

	Perceived Change in the Individual Financial Situation since the COVID-19 Pandemic		Perceived Worse Financial Situation			
	Got Worse *n* (%)	The Same as Before/Got Better *n* (%)			Adjusted OR (CI 95%)	*p* Value
Total	569 (55.6)	454 (44.4)				
Sex						
Women	316 (59.6)	214 (40.4)			1.13 (0.87–1.48)	0.379
Men	252 (51.2)	240 (48.8)			1	
Age						
18–25	74 (47.4)	82 (52.6)			1	
26–45	381 (55.5)	305 (44.5)			1.63 (1.12–2.37)	0.011
>45	112 (62.6)	67 (37.4)			2.01 (1.25–3.25)	0.004
Education level						
Basic education	155 (57.4)	115 (42.6)			0.92 (0.64–1.32)	0.656
Secondary education	246 (56.6)	189 (43.4)			1.02 (0.75–1.41)	0.871
Higher education	168 (53.8)	144 (46.2)			1	
Monthly income before COVID-19 outbreak						
<EUR 650	415 (65.0)	223 (35.0)			2.81 (2.13–3.72)	<0.001
≥EUR 650	143 (39.4)	220 (60.6)			1	
Length of stay in Portugal						
<1 year	118 (60.8)	76 (39.2)			1.45 (0.91–2.31)	0.121
1 to 5 years	374 (55.4)	301 (44.6)			1.24 (0.85–1.81)	0.273
≥6 years	77 (50.1)	75 (49.9)			1	
Migration status						
Documented/in regularization	501 (54.4)	420 (45.6)			1	
Undocumented	59 (67.0)	29 (33.0)			1.43 (0.88–2.38)	0.155

**Table 3:** Characteristics of the participants by perceived change in their individual health condition since the COVID-19 pandemic and factors associated with worse health condition.

	Perceived Change in the Individual Health Condition since the COVID-19 Pandemic		Perceived Worse Health Condition		
	Got Worse *n* (%)	The Same as Before/Got Better *n* (%)		Adjusted OR (CI 95%)	*p* Value
Total	208 (19.9)	836 (80.1)			
Sex					
Women	134 (24.4)	416 (75.6)		1.58 (1.13–2.20)	0.007
Men	73 (14.8)	420 (85.2)		1	
Age					
18–25	28 (16.6)	141 (83.4)		1	
26–45	130 (18.9)	559 (81.1)		1.31 (0.82–2.15)	0.261
>45	50 (27.2)	134 (72.8)		1.78 (1.02–3.16)	0.044
Education level					
Basic education	71 (25.1)	212 (74.9)		1.57 (1.01–2.47)	0.047
Secondary education	90 (20.2)	356 (79.8)		1.51 (1.01–2.29)	0.049
Higher education	46 (15.0)	261 (85.0)		1	
Monthly income before COVID-19 outbreak					
<EUR 650	153 (23.3)	503 (76.7)		1.69 (1.18–2.44)	0.005
≥EUR 650	50 (13.7)	314 (86.3)		1	
Length of stay in Portugal					
<1 year	38 (18.4)	168 (81.6)		1	
1 to 5 years	135 (19.8)	548 (80.2)		1.12 (0.80–1.88)	0.378
≥6 years	35 (22.9)	118 (77.1)		1.44 (0.82–2.51)	0.203
Migration status					
Documented/in regularization	182 (19.4)	754 (80.6)		1	
Undocumented	22 (23.4)	72 (76.6)		1.07 (0.61–1.80)	0.817

## Data Availability

The data presented in this study are available on request from the corresponding author. The data are not publicly available due to confidentiality reasons.

## Supplementary Materials

The following supporting information can be downloaded at: https://www.mdpi.com/article/10.3390/ijerph19031786/s1, Table S1: Univariate analysis of the factors associated with worse financial situation.
