# Supplementary material for: How Did the COVID-19 Pandemic Affect Migrant Populations in Lisbon, Portugal? A Study on Perceived Effects on Health and Economic Condition
Source: Int J Environ Res Public Health. 2022 Feb 4;19(3):1786. doi: 10.3390/ijerph19031786 (PMC8835335; doi:10.3390/ijerph19031786)
Supplement: Supplementary file 1 [file ijerph-19-01786-s001.zip › ijerph-1505053-supplementary.pdf]

Supplementary file

Table S1. Univariate analysis of the factors associated with worse financial situation.

	Worse Financial Situation	
	Crude OR (CI 95%)	*p-value*
Total		
Sex		
Women	1.41 (1.10–1.80)	*0.007*
Men	1	
Age		
16–25	1	
26–45	1.38 (0.98–1.96)	*0.067*
>45	1.85 (1.20–2.87)	*0.006*
Education level		
Basic education	1.16 (0.83–1.61)	*0.390*
Secondary education	1.12 (0.83–1.49)	*0.460*
Higher education	1	
Monthly household income		
<650€	2.86 (2.20–3.74)	*<0.001*
≥650€	1	
Length of stay in Portugal		
<1 year	1.51 (0.99–2.33)	*0.059*
1 to 5 years	1.21 (0.85–1.72)	*0.288*
≥6 years	1	
Migration status		
Documented/in regularization	1	
Undocumented	1.71 (1.08–2.74)	*0.023*
